# Supplementary material for: Healthcare provider and patient/family perceptions of continuous pressure imaging technology for prevention of pressure injuries: A secondary analysis of patients enrolled in a randomized control trial
Source: PLoS One. 2022 Nov 29;17(11):e0278019. doi: 10.1371/journal.pone.0278019 (PMC9707747; doi:10.1371/journal.pone.0278019)
Supplement: S1 File — (PDF) [file pone.0278019.s001.pdf]

Healthcare Provider Perceptions of a Pressure-Sensing Mattress Cover System

If you have had a patient in the XSensor study, then please complete this quick 5 minute survey. Your responses to the survey are an important part of our research study because it will help us to understand what you thought about using the system and its impact on the care of your patients. We really appreciate your involvement and support, and we thank you for making this study possible.

1. Please specify your profession?
    a. Nurse
    b. Healthcare aid
    c. Clinician
    d. Other: ____

2. Have you used any pressure-sensing mattress cover system, such as XSENSOR's ForeSite PT System, prior to this study?
    a. Yes
    b. No

2a. Which unit did you use the pressure-sensing mattress system for this study?
    a. Unit 36
    b. Unit 37
    c. Unit 100
    d. Unit 101
    e. Unit 112
    f. ICU

3. Please answer the following by circling one of the numbered choices:

	Strongly Disagree	Disagree	Neither Disagree or Agree	Agree	Strongly Agree
Overall, the pressure-sensing mattress system was useful for me.	1	2	3	4	5
Overall, the pressure-sensing mattress system was easy to use.	1	2	3	4	5
Information provided by the pressure-sensing mattress system was easy to understand	1	2	3	4	5
You were able to identify high pressure area(s) on the patient.	1	2	3	4	5
Information provided by the system helped me in knowing how to re-position the patient.	1	2	3	4	5
Information provided by the pressure-sensing mattress system corresponds with routine assessments for risk for developing pressure ulcers (e.g. Braden scale)	1	2	3	4	5
Use of the pressure-sensing mattress system positively affected the way you provide pressure relief	1	2	3	4	5
Use of the pressure-sensing mattress system positively impacted (improved) patient care.	1	2	3	4	5

4. Check all the features that you liked about the system?
   - ☐ 'Reset Turn Timer' button
   - ☐ 'Patient Turn Timer' countdown
   - ☐ Patient Display Area
   - ☐ 'Recent Turns and Pressure Exposure' bar graph
   - ☐ 'Help' button
   - ☐ 'Options' button
   - ☐ 'Previous Turns' information box
   - ☐ 'Exposure indicator(s)'
   - ☐ Changing the 'Turn Timer Interval'
   - ☐ Changing the brightness
   - ☐ Other: ____________________________
     ____

5. What features did you dislike about the system?

6. What are the advantages of using the pressure-sensing mattress cover system?

7. What are the disadvantages of using the pressure-sensing mattress cover system?

8. Please explain any differences on the impact on patient care from the use of the pressure-sensing mattress cover system compared to not using the system?

9. How would you improve the pressure-sensing mattress cover system (e.g. design, information to include, location of monitor, use of 'patient turn' button, etc.)?

10. Additional comments:

Thank you for completing this survey! Your responses will remain anonymous.
