# Supplementary material for: Healthcare provider and patient/family perceptions of continuous pressure imaging technology for prevention of pressure injuries: A secondary analysis of patients enrolled in a randomized control trial
Source: PLoS One. 2022 Nov 29;17(11):e0278019. doi: 10.1371/journal.pone.0278019 (PMC9707747; doi:10.1371/journal.pone.0278019)
Supplement: S2 File — (PDF) [file pone.0278019.s002.pdf]

Record ID: ______________________________

Patient Perceptions to a Pressure-Sensing Mattress System

Thank you for participating in this study. Please take 5 minutes to complete this survey, which will help our research team understand what you thought about the pressure-sensing mattress system, and how it influenced care provided by healthcare providers.

1. Specify the person filling out this form.
   - ☐ Patient
   - ☐ Proxy: ___________________________

2. Has a pressure-sensing mattress system been used for you/the patient before?
   - ☐ Yes
   - ☐ No

3. Please answer the following by checking one of the numbered choices:

	Strongly Disagree	Disagree	Neither Disagree or Agree	Agree	Strongly Agree
The sensor mat was comfortable to lie/sleep on.	1 ☐	2 ☐	3 ☐	4 ☐	5 ☐
The sensor mat moved significantly when laid/slept on.	1 ☐	2 ☐	3 ☐	4 ☐	5 ☐
The pressure relief provided by the nurse was positively influenced by the system	1 ☐	2 ☐	3 ☐	4 ☐	5 ☐

4. Did you or the patient request that the sensor mat be removed?
   - ☐ Yes
   - ☐ No

5. Did you or the patient request that the LCD monitor be turned off?
   - ☐ Yes
   - ☐ No

Record ID: ______________________________

6. Additional comments:
____
____
____
