# Supplementary material for: Healthcare provider and patient/family perceptions of continuous pressure imaging technology for prevention of pressure injuries: A secondary analysis of patients enrolled in a randomized control trial
Source: PLoS One. 2022 Nov 29;17(11):e0278019. doi: 10.1371/journal.pone.0278019 (PMC9707747; doi:10.1371/journal.pone.0278019)
Supplement: Attachment — Submitted filename: Evaluation-11 avril 2022.docx [file pone.0278019.s003.docx]

The subject addressed by this paper, namely the perceptions of healthcare providers, patients, and family members in understanding the acceptability of continuous pressure imaging (CPI) technology, is important and still relevant today. Here are some comments to improve the paper.

**Major comments**

1. In the section **Sampling and subject recruitment** authors wrote: “*The control group had the ForeSite PT™ system turned on for collecting continuous interface pressure data but the LCD monitor was covered so as not to provide visual feedback to the healthcare providers*” What precautions have been taken so that caregivers do not seek to watch the information for the good of their patients?

In this section, authors should indicate and justify their sample size.

2. In the section of **Content of Surveys** authors should indicate the level of measure of their questions and then indicated if yes or not their data collection tools was validated one.

3. Authors wrote « *Closed-ended questions were analyzed using inductive thematic analysis with descriptive statistics using Microsoft Excel* » If one can understand that opened-ended questions will be analyse using inductive thematic, however, it seems difficult to apply this to closed-ended questions administrated to more than 125 participants and specially with question on a Likert scale. So, it will be better for authors to describe separately how the analysed qualitative data and quantitative one.

4. Authors wrote “*The intervention group had the ForeSite PT™ system set up with the monitor displayed for healthcare workers to view. The control group had the ForeSite PT™ system turned on for collecting continuous interface pressure data but the LCD monitor was covered so as not to provide visual feedback to the healthcare providers. In this case, the healthcare worker provided standard of care to the patient without visual feedback on pressure recordings*” This is good, but in following sections such us Analysis section or Results section authors did not refer to intervention group and control group. If they used case control study design they should rewrite analysis section and show how they compare this two groups and, in their results sections, they should used adequate statistics to analysed quantitative data.

If they used another design, so, they should include a study design section and them describe it and adjusted they other section to it.

5. Beware of the desirability bias of patients and healthcare professionals. How was this handled?
