# Supplementary material for: Healthcare provider and patient/family perceptions of continuous pressure imaging technology for prevention of pressure injuries: A secondary analysis of patients enrolled in a randomized control trial
Source: PLoS One. 2022 Nov 29;17(11):e0278019. doi: 10.1371/journal.pone.0278019 (PMC9707747; doi:10.1371/journal.pone.0278019)
Supplement: Attachment — Submitted filename: Response to Reviewers June 23, 2022.docx [file pone.0278019.s004.docx]

Wrechelle Ocampo

Cumming School of Medicine

University of Calgary
3330 Hospital Drive NW 
Calgary, Alberta T2N 4N1

Solna Carreon Santos

Editorial Manager

PLOS ONE

San Francisco, California

June 23, 2022

Dear Dr. Carreon Santos,

I am writing to inform you that I have addressed the issues that I was informed of. I updated the manuscript with the requested revision to insert the ethics statement at the beginning of the Methods section. The statement has also been added to the “Ethics Statement” field of the submission form.

Regarding the Data Availability Statement, we are requesting that data are available upon request for legal restrictions based on University of Calgary’s policies on sharing research data to external researchers. I have updated the statement to reflect this reasoning.

Thank you for your time and consideration of our revised manuscript. We look forward to your response.

Kind regards,

Wrechelle Ocampo

Wrechelle Ocampo, MBT

Project Coordinator, W21C Research and Innovation Centre

O’Brien Institute for Public Health, Cumming School of Medicine, University of Calgary
