# Supplementary material for: Healthcare provider and patient/family perceptions of continuous pressure imaging technology for prevention of pressure injuries: A secondary analysis of patients enrolled in a randomized control trial
Source: PLoS One. 2022 Nov 29;17(11):e0278019. doi: 10.1371/journal.pone.0278019 (PMC9707747; doi:10.1371/journal.pone.0278019)
Supplement: Attachment — Submitted filename: Commentaire reevaluation 22 juillet 2022.docx [file pone.0278019.s005.docx]

**The authors have responded to all comments appropriately. However, a new comment requires a response before the paper is published.**

In the Sampling and Subject Recruitment, authors wrote: “For the RCT, the calculated required sample size was based on the assumption of a 33% relative risk reduction in pressure injuries, an alpha level of 0.05 and 80% power. This led to an anticipated need for 678 randomized patients with an expected attrition proportion of 12% (12)”

And in the results section, under Patient/Family Survey sub-section, they wrote: “A total of 525 respondents completed this survey. There were 154 patients/family members who were unable to complete the survey either due to the death of the patient or being discharged from the unit before the survey could be completed.”

So as the sample size is less than the expected one, authors should discuss the implications of that on their results obtained.
