# Supplementary material for: Healthcare provider and patient/family perceptions of continuous pressure imaging technology for prevention of pressure injuries: A secondary analysis of patients enrolled in a randomized control trial
Source: PLoS One. 2022 Nov 29;17(11):e0278019. doi: 10.1371/journal.pone.0278019 (PMC9707747; doi:10.1371/journal.pone.0278019)
Supplement: Attachment — Submitted filename: Response to Reviewers Oct 13, 2022.docx [file pone.0278019.s006.docx]

Wrechelle Ocampo

Cumming School of Medicine

University of Calgary
3330 Hospital Drive NW 
Calgary, Alberta T2N 4N1

Yih-Kuen Jan

Academic Editor

PLOS ONE

San Francisco, California

October 12, 2022

Dear Dr. Jan,

I am writing in response to the requested revisions for manuscript PONE-D-21-31308, “Healthcare provider and patient perceptions of continuous pressure imaging technology for prevention of pressure injuries: a secondary analysis of patients enrolled in a randomized control trial.” I have updated the manuscript with the requested revisions and have responded to both reviewer’s comments below, which I thank them for providing.

**Reviewer #1:** The authors have responded to all comments appropriately. However, a new comment requires a response before the paper is published. In the Sampling and Subject Recruitment, authors wrote: “For the RCT, the calculated required sample size was based on the assumption of a 33% relative risk reduction in pressure injuries, an alpha level of 0.05 and 80% power. This led to an anticipated need for 678 randomized patients with an expected attrition proportion of 12% (12).” And in the results section, under Patient/Family Survey sub-section, they wrote: “A total of 525 respondents completed this survey. There were 154 patients/family members who were unable to complete the survey either due to the death of the patient or being discharged from the unit before the survey could be completed.” So as the sample size is less than the expected one, authors should discuss the implications of that on their results obtained.

Response: The sample size of 678 was determined for the primary outcome. Not all 678 participants responded to the survey. The manuscript with tracked changes includes the following revision on page 6, lines 95-98: “This led to an anticipated need for 678 randomized patients with an expected attrition proportion of 12% for the primary study outcome (12). In a secondary exploratory analysis, a convenience sample made up of eligible hospitalized patients (or family members) and healthcare providers was used to evaluate perceptions about CPI.”

**Reviewer #2:**

General comments: The study aimed to report on the opinions of healthcare providers and patients or family members about CPI.

The study is good to survey the patients and healthcare providers in pressure sensing devices. But, is there any intellectual property (ForeSite PT) to the study?
The researchers may figure out how participants were collected, eliminated and included in the RCT study.

Response: There is no intellectual property as this study was done independently from XSENSOR Technology Corporation.

Specific Comment 1: Line 72: “The main study was a RCT that assessed the effect of the ForeSite PT™ system”

\## Might researchers mention the instrument completely (company, state, country)? ## The “a RCT” should be changed into “an RCT.”

Response: The grammar of the sentence was corrected and the company was added on page 5, lines 77-78 of the revised manuscript with tracked changes: “The main study was an RCT that assessed the effect of the ForeSite PT™ system (XSESNSOR Technology Corp., Calgary, AB, Canada)”

Specific Comment 2: Line 73-74: “The system consisted of two parts: a flexible, thin mattress cover with embedded sensors that was positioned under a fitted hospital sheet”
\## How do researchers know that the devices have been calibrated (in good condition)?

Response: XSENSOR Tech Support did a system upgrade before recruitment initiation and half-way through the study. This was added to page 6, lines 88-89 of the revised manuscript with tracked changes: “Systems had software updates by the developer prior to initiation of study recruitment and half-way through study recruitment.”

Specific Comment 3: Line 96-99: “The control group had the ForeSite PT™ system turned on for collecting continuous interface pressure data but the LCD monitor was covered, and the settings were adjusted to low sensitivity, such that the visual feedback to the healthcare providers even, if the monitor was turned on, would not highlight areas of the body in need of pressure relief”
\## In this case, how the researchers did “benefit and harm” principles in bioethics?

Response: Standard of care was still provided in the control group, so there was no harm to the patient. The benefits of the intervention over the control were explained when patients were consented. This has been reflected on pages 6-7, lines 107-110: “For control patients, the healthcare worker provided standard of care to the patient without visual feedback on pressure recordings. During the process of informed consent, the benefit of the intervention over standard of care and randomization to either group was explained to the participant or surrogate.”

Specific Comment 4: Line 106: “Content of Surveys”.
\## How did the researchers validity and reliability of the questionnaire?

Response: I added information that pertains to the validity and reliability of the questionnaire on pages 7-8, lines 127-131 of the manuscript with tracked changes: “. Contact information of the study team was provided in the email invitation to the survey in case healthcare providers had questions, or the study team was available to answer questions when the survey was distributed in-person. There were no concerns or questions about the surveys by either group, and respondents were not obligated to answer questions that they were uncomfortable answering.”

Specific Comment 5: Line 220-221: “Nearly a fifth of healthcare providers had used the CPI technology before. This prior use of the device may have influenced their responses on the use of the device in this study.”
\## Why have researchers not excluded the participants (nurses) that have been familiar with the devices before?

Response: Providers may have used other models of CPI and not necessarily the ForeSite PT System. Since this aspect of the study was exploratory, we wanted to evaluate as much of the collected data as possible. The intent of the question was to get a sense of familiarity with CPI technology. The manuscript was updated on page 16, lines 230-231 of the tracked changes version: “This prior use of the device may have influenced their responses on the use of the device in this study with an unconscious bias.”

Specific Comment 6: For bedsore monitoring to prevent pressure injuries, the authors may consider citing the article below.
\## Analysis of Moisture and Sebum of the Skin for Monitoring Wound Healing in Older Nursing Home Residents. In: Vol. 1215 AISC. Advances in Intelligent Systems and Computing (pp. 177-182), 2020
\## A time motion study of manual versus artificial intelligence methods for wound assessment, Plos one, 2022.

Response: Thank you for the interesting references, but I feel that they are just outside the scope of this paper.

Thank you for your time and consideration of our revised manuscript. We look forward to your response.

Kind regards,

Wrechelle Ocampo

Wrechelle Ocampo, MBT

Project Coordinator, W21C Research and Innovation Centre

O’Brien Institute for Public Health, Cumming School of Medicine, University of Calgary
